# Supplementary material for: On-Treatment Albumin-Bilirubin Grade: Predictor of Response and Outcome of Sorafenib-Regorafenib Sequential Therapy in Patients with Unresectable Hepatocellular Carcinoma
Source: Cancers (Basel). 2021 Jul 26;13(15):3758. doi: 10.3390/cancers13153758 (PMC8345148; doi:10.3390/cancers13153758)
Supplement: Supplementary file 1 [file cancers-13-03758-s001.zip › cancers-1270378-supplementary.pdf]

cancers

MDPI

Article

On-Treatment Albumin–Bilirubin Grade: Predictor of Response and Outcome of Sorafenib–Regorafenib Sequential Therapy in Patients with Unresectable Hepatocellular Carcinoma

Hung-Wei Wang, Po-Heng Chuang, Wen-Pang Su, Jung-Ta Kao, Wei-Fan Hsu, Chun-Che Lin, Guan-Tarn Huang, Jaw-Town Lin, Hsueh-Chou Lai and Cheng-Yuan Peng

Supplementary Materials:

Table S1. Liver reserve during sequential regorafenib treatment.

Regorafenib	1st round	2nd round	3rd round	4th round	5th round
**Total *n***	88	53	38	24	14
**Child-Pugh class A**	84	50	34	22	12
**A5**	60	38	26	17	9
**A6**	24	12	8	5	3
**Child-Pugh class B**	4	3	4	2	2
**Stop due to PD (%)**	NA	30/35 (85.7)	13/15 (86.7)	14/14 (100)	10/10 (100)
**Rate of keeping Child-Pugh class A (%)**	NA	50/84 (59.5)	34/50 (68)	22/34 (64.7)	12/22 (54.5)

Abbreviations: NA, not available; PD, progressive disease.

Table S2. Characteristics of patient cohorts receiving and not receiving sequential therapy (regorafenib) after sorafenib treatment failure.

**Variables Median ± IQR or *n* (%)**		**Before propensity score matching**			**After propensity score matching**		
		**Without sequential therapy (*n* = 90)**	**With sequential therapy (*n* = 88)**	***p* value**	**Without sequential therapy (*n* = 43)**	**With sequential therapy (*n* = 43)**	***p* value**
Age (year)		61 ± 17	66 ± 14	0.005	61 ± 20	63 ± 13	0.565
Sex	Male/female	73/17	69/19	0.654	8/35	9/34	0.787
Etiology	HBV/HCV/ HBV+HCV/NBNC	41/20/4/25	45/28/2/13	0.114	18/12/2/11	26/13/0/4	0.080
ECOG PS	0/1/2/3	34/30/25/1	75/10/3/0	< 0.001	32/7/4/0	34/6/3/0	0.869
Child-Pugh score	5/6/≥ 7	38/47/5	59/24/5	0.002	24/17/2	31/10/2	0.138
Child-Pugh class	A/B	85/5	83/5	0.971	41/2	41/2	1.000
ALBI score		-2.35 ± 0.70	-2.64 ± 0.57	< 0.001	-2.51 ± 0.71	-2.57 ± 0.60	0.323
ALBI grade	1/2/3	28/60/2	48/40/0	0.002	20/23	21/22	0.829
FIB-4		3.70 ± 4.60	3.59 ± 3.13	0.224	3.87 ± 4.12	3.62 ± 2.65	0.455
FIB-4	< 3.25/≥ 3.25	38/52	31/45	0.852	17/26	16/27	0.825
BCLC stage	A/B/C	0/5/85	1/23/64	< 0.001	0/5/38	0/7/36	0.534
MVI	presence	38 (42.2)	28 (31.8)	0.151	19 (44.2)	16 (37.2)	0.510
EHS	presence	68 (75.6)	51 (58)	0.013	29 (67.4)	29 (67.4)	1.000
AFP (ng/mL)		291.4 ± 3822.5	17.3 ± 292.4	0.001	134.3 ± 2906.1	19.4 ± 1223.2	0.260
AFP (ng/mL)	< 400/≥ 400	48/42	66/21	0.002	17 (39.5)	15 (34.9)	0.655
Albumin (g/dL)		3.7 ± 0.7	4.0 ± 0.7	< 0.001	3.9 ± 0.7	4.0 ± 0.8	0.438
AST (U/L)		56 ± 46	39 ± 21	< 0.001	53 ± 37	46 ± 22	0.073
ALT (U/L)		44 ± 41	31 ± 22	< 0.001	41 ± 31	31 ± 29	0.137
Total bilirubin (mg/dL)		1.1 ± 0.5	0.8 ± 0.5	< 0.001	1.0 ± 0.5	0.8 ± 0.5	0.234
INR		1.10 ± 0.20	1.05 ± 0.10	< 0.001	1.09 ± 0.15	1.05 ± 0.12	0.071
TKI duration (months)		4.4 ± 7.5	2.8 ± 3.4	0.032	5.0 ± 6.0	6.2 ± 11.1	0.068
Locoregional therapy after sorafenib failure	Yes (RFA/TACE/RT/ Combination)	51 (56.7) (2/17/17/15)	49 (55.7) (1/40/1/7)	0.881	30 (69.8) (2/11/7/10)	26 (60.5) (1/19/1/5)	0.367

Abbreviations: AFP—alpha-fetoprotein; ALBI—albumin–bilirubin; ALT—alanine aminotransferase; AST—aspartate aminotransferase; BCLC—Barcelona Clinic Liver Cancer staging; ECOG PS—Eastern Cooperative Oncology Group performance status; EHS—extrahepatic spread; FIB-4—fibrosis index based on four factors; HBV—hepatitis B virus;

HCV—hepatitis C virus; INR—international normalized ratio; IQR—interquartile range; MVI—macrovascular invasion; NBNC—non–hepatitis B and non–hepatitis C; RFA—radiofrequency ablation; RT—radiotherapy; TACE—transarterial chemoembolization; TKI—tyrosine kinase inhibitor.

Table S3. Results of univariate and multivariate Cox regression analyses of predictors of PFS after regorafenib therapy.

			Child-Pugh score-based Model		ALBI grade-based Model	
**Variables**	**Univariate Hazard Ratio (95% CI)**	***p* value**	**Multivariate Hazard Ratio (95% CI)**	***p* value**	**Multivariate Hazard Ratio (95% CI)**	***p* value**
Age (year)	1.009 (0.985–1.033)	0.471				
Male vs female	1.598 (0.834–3.060)	0.158	1.664 (0.841–3.291)	0.144	2.046 (1.050–3.984)	0.035
Child-Pugh score 5 vs 6	0.571 (0.333–0.979)	0.042	0.579 (0.335–1.003)	0.051		
ALBI grade 1 vs 2	0.519 (0.317–0.850)	0.009			0.432 (0.258–0.722)	0.001
FIB-4 < 3.25 vs ≥ 3.25	0.795 (0.435–1.451)	0.454				
BCLC stage B vs C	1.050 (0.595–1.855)	0.866				
MVI (no vs yes)	0.813 (0.490–1.349)	0.423				
EHS (no vs yes)	0.946 (0.573–1.561)	0.827				
AFP < 20 vs ≥ 20 (ng/mL)	0.619 (0.377–1.016)	0.058	0.632 (0.379–1.055)	0.079	0.556 (0.337–0.919)	0.022
ALBI grade 2 and AFP ≥ 20 ng/mL (yes vs no)	2.765 (1.538–4.973)	0.001				
Albumin (g/dL)	0.635 (0.369–1.092)	0.101				
AST (U/L)	1.009 (1.000–1.018)	0.041				
ALT (U/L)	1.002 (0.995–1.009)	0.572				
Total bilirubin (mg/dL)	0.895 (0.444–1.805)	0.757				
PLT (10^9/L)	1.002 (0.999–1.005)	0.194				
INR	0.633 (0.021–18.93)	0.792				

Table shading indicated that the variable has a confounding effect on other factors, and thus was not included in the multivariate analysis. Abbreviations: AFP—alpha-fetoprotein; ALBI—albumin–bilirubin; ALT—alanine aminotransferase; AST—aspartate aminotransferase; BCLC—Barcelona Clinic Liver Cancer staging; CI—confidence interval; EHS—extrahepatic spread; FIB-4—fibrosis index based on four factors; INR—international normalized ratio; MVI—macrovascular invasion; PFS—progression-free survival; PLT—platelets.

Table S4. Results of univariate and multivariate Cox regression analyses of predictors of OS after regorafenib therapy.

			Child-Pugh score-based Model		ALBI grade-based Model	
**Variables**	**Univariate Hazard Ratio (95% CI)**	***p* value**	**Multivariate Hazard Ratio (95% CI)**	***p* value**	**Multivariate Hazard Ratio (95% CI)**	***p* value**
Age (year)	1.003 (0.961–1.047)	0.888				
Male vs female	2.369 (0.547–10.26)	0.249	2.198 (0.485–9.960)	0.307	2.643 (0.584–11.95)	0.207
Child-Pugh score 5 vs 6	0.465 (0.183–1.179)	0.107	0.681 (0.206–2.249)	0.529		
ALBI grade 1 vs 2	0.511 (0.205–1.270)	0.148			0.543 (0.190–1.556)	0.256
FIB-4 < 3.25 vs ≥ 3.25	0.329 (0.092–1.182)	0.088	0.545 (0.167–1.775)	0.314	0.514 (0.158–1.671)	0.269
BCLC stage B vs C	0.543 (0.158–1.864)	0.332				
MVI (no vs yes)	0.768 (0.302–1.953)	0.579				
EHS (no vs yes)	0.875 (0.344–2.227)	0.779				
AFP < 20 vs ≥ 20 (ng/mL)	0.301 (0.112–0.809)	0.017	0.314 (0.102–0.968)	0.044	0.322 (0.109–0.946)	0.039
ALBI grade 2 and AFP ≥ 20 ng/mL (yes vs no)	4.620 (1.737–12.29)	0.002				
Albumin (g/dL)	0.587 (0.231–1.493)	0.263				
AST (U/L)	1.012 (0.996–1.028)	0.149				
ALT (U/L)	1.004 (0.990–1.018)	0.575				
Total bilirubin (mg/dL)	1.177 (0.308–4.497)	0.812				
PLT (10^9/L)	1.000 (0.996–1.004)	0.950				
INR	2.934 (0.006–1560)	0.737				

Table shading indicated that the variable has a confounding effect on other factors, and thus was not included in the multivariate analysis. Abbreviations: AFP—alpha-fetoprotein; ALBI—albumin–bilirubin; ALT—alanine aminotransferase; AST—aspartate aminotransferase; BCLC—Barcelona Clinic Liver Cancer staging; CI—confidence interval; EHS—extrahepatic spread; FIB-4—fibrosis index based on four factors; INR—international normalized ratio; MVI—macrovascular invasion; OS—overall survival; PLT—platelets.

Table S5. Results of univariate and multivariate Cox regression analyses of predictors of OS for sorafenib–regorafenib sequential therapy.

Variables	Univariate Hazard Ratio (95% CI)	*p* value	Child-Pugh score-based Model: Multivariate Hazard Ratio (95% CI)	*p* value	ALBI grade-based Model: Multivariate Hazard Ratio (95% CI)	*p* value
Age (year)	1.018 (0.977–1.060)	0.400				
Male vs female	1.713 (0.391–7.498)	0.475				
Child-Pugh score 5 vs 6	1.736 (0.684–4.405)	0.246	0.986 (0.289–3.361)	0.982		
ALBI grade 1 vs 2	0.386 (0.154–0.970)	0.043			0.303 (0.112–0.821)	0.019
FIB-4 < 3.25 vs ≥ 3.25	0.376 (0.105–1.351)	0.134	0.501 (0.148–1.700)	0.268	0.548 (0.161–1.864)	0.335
BCLC stage B vs C	0.431 (0.125–1.487)	0.183	0.698 (0.113–4.298)	0.699	0.612 (0.133–2.814)	0.528
MVI (no vs yes)	0.961 (0.377–2.445)	0.933				
EHS (no vs yes)	0.570 (0.223–1.460)	0.241	0.818 (0.172–3.901)	0.801	0.705 (0.205–2.421)	0.579
AFP < 20 vs ≥ 20 (ng/mL)	0.321 (0.120–0.859)	0.024	0.400 (0.129–1.238)	0.112	0.335 (0.122–0.919)	0.034
ALBI grade 2 and AFP ≥ 20 ng/mL (yes vs no)	5.198 (1.898–14.24)	0.001				
Albumin (g/dL)	0.466 (0.176–1.232)	0.124				
AST (U/L)	1.017 (0.998–1.037)	0.079				
ALT (U/L)	1.009 (0.994–1.024)	0.245				
Total bilirubin (mg/dL)	1.215 (0.293–5.026)	0.789				
PLT (10^9/L)	1.001 (0.996–1.005)	0.768				

Table shading indicated that the variable has a confounding effect on other factors, and thus was not included in the multivariate analysis. Abbreviations: AFP—alpha-fetoprotein; ALBI—albumin–bilirubin; ALT—alanine aminotransferase; AST—aspartate aminotransferase; BCLC—Barcelona Clinic Liver Cancer staging; CI—confidence interval; EHS—extrahepatic spread; FIB-4—fibrosis index based on four factors; INR—international normalized ratio; MVI—macrovascular invasion; OS—overall survival; PLT—platelets.

Table S6. Results of univariate and multivariate logistic regression analyses of predictors of objective response (CR and PR) after regorafenib therapy.

			Child-Pugh score-based Model		ALBI grade-based Model	
**Variables**	**Univariate Odds Ratio (95% CI)**	***p* value**	**Multivariate Odds Ratio (95% CI)**	***p* value**	**Multivariate Odds Ratio (95% CI)**	***p* value**
Age (year)	0.988 (0.944–1.035)	0.616				
Male vs female	0.533 (0.158–1.800)	0.311				
Child-Pugh score 5 vs 6	1.871 (0.477–7.333)	0.369				
ALBI grade 1 vs 2	1.528 (0.505–4.619)	0.453				
FIB-4 < 3.25 vs ≥ 3.25	2.842 (0.747–10.81)	0.125	1.799 (0.487–6.643)	0.378	2.702 (0.628–11.62)	0.182
BCLC stage B vs C	2.018 (0.663–6.137)	0.216	1.260 (0.324–4.900)	0.739	0.738 (0.153–3.559)	0.705
MVI (no vs yes)	4.286 (0.902–20.36)	0.067	5.935 (0.620–56.81)	0.122	10.47 (0.883–124.2)	0.063
EHS (no vs yes)	1.625 (0.557–4.744)	0.374				
AFP < 20 vs ≥ 20 (ng/mL)	3.059 (0.903–10.36)	0.073	2.342 (0.620–8.851)	0.210	2.126 (0.546–8.288)	0.277
Albumin (g/dL)	0.749 (0.226–2.484)	0.636				
AST (U/L)	0.975 (0.935–1.016)	0.225				
ALT (U/L)	0.967 (0.926–1.010)	0.127				
Total bilirubin (mg/dL)	2.246 (0.454–11.11)	0.321				
PLT (10^9/L)	1.001 (0.996–1.006)	0.666				
INR	472.6 (0.158–1409322)	0.131			12713 (0.070–2301371285)	0.126
HFSR	1.610 (0.536–4.833)	0.396				

Table shading indicated that the variable has a confounding effect on other factors, and thus was not included in the multivariate analysis. Abbreviations: AFP—alpha-fetoprotein; ALBI—albumin–bilirubin; ALT—alanine aminotransferase; AST—aspartate aminotransferase; BCLC—Barcelona Clinic Liver Cancer staging; CI—confidence interval; CR—complete response; EHS—extrahepatic spread; FIB-4—fibrosis index based on four factors; HFSR—hand–foot skin reaction; INR—international normalized ratio; MVI— macrovascular invasion; PLT—platelets; PR—partial response.

Table S7. Results of univariate and multivariate logistic regression analyses of predictors of disease control (CR, PR, and SD) after regorafenib therapy.

			Child-Pugh score-based Model		ALBI grade-based Model	
**Variables**	**Univariate Odds Ratio (95% CI)**	***p* value**	**Multivariate Odds Ratio (95% CI)**	***p* value**	**Multivariate Odds Ratio (95% CI)**	***p* value**
Age (year)	0.967 (0.930–1.007)	0.104				
Male vs female	0.369 (0.117–1.165)	0.089	0.415 (0.113–1.516)	0.183	0.222 (0.053–0.928)	0.039
Child-Pugh score 5 vs 6	2.406 (0.872–6.642)	0.090	1.461 (0.448–4.759)	0.529		
ALBI grade 1 vs 2	3.875 (1.546–9.712)	0.004			5.061 (1.602–15.99)	0.006
FIB-4 < 3.25 vs ≥ 3.25	2.133 (0.735–6.195)	0.164	2.466 (0.823–7.392)	0.107	2.631 (0.850–8.139)	0.093
BCLC stage B vs C	1.142 (0.441–2.958)	0.784				
MVI (no vs yes)	1.391 (0.548–3.528)	0.487				
EHS (no vs yes)	1.071 (0.449–2.554)	0.877				
AFP < 20 vs ≥ 20 (ng/mL)	1.384 (0.579–3.307)	0.465				
Albumin (g/dL)	3.847 (1.260–11.75)	0.018				
AST (U/L)	0.971 (0.943–1.001)	0.059				
ALT (U/L)	0.986 (0.965–1.008)	0.203				
Total bilirubin (mg/dL)	1.264 (0.328–4.873)	0.733				
PLT (10^9/L)	0.998 (0.994–1.003)	0.452				
INR	0.341 (0.000–280.3)	0.753				
HFSR	2.480 (0.951–6.464)	0.063	3.113 (1.003–9.659)	0.049	3.360 (0.980–11.52)	0.054

Table shading indicated that the variable has a confounding effect on other factors, and thus was not included in the multivariate analysis. Abbreviations: AFP—alpha-fetoprotein; ALBI—albumin–bilirubin; ALT—alanine aminotransferase; AST—aspartate aminotransferase; BCLC—Barcelona Clinic Liver Cancer staging; CI—confidence interval; CR—complete response; EHS—extrahepatic spread; FIB-4—fibrosis index based on four factors; HFSR—hand foot skin reaction; INR—international normalized ratio; MVI— macrovascular invasion; PLT—platelets; PR—partial response; SD—stable disease.

Table S8. Results of univariate and multivariate Cox regression analyses of predictors of OS after failure of sorafenib therapy (all patients, *n* = 178).

Patients with and without regorafenib therapy after failure of sorafenib therapy (*n* = 178)						
			ALBI grade-based Model		Combined ALBI and AFP-based Model	
**Variables**	**Univariate Hazard Ratio (95% CI)**	***p* value**	**Multivariate Hazard Ratio (95% CI)**	***p* value**	**Multivariate Hazard Ratio (95% CI)**	***p* value**
Age (year)	0.984 (0.966–1.002)	0.075				
Male vs female	1.155 (0.703–1.899)	0.570				
ALBI grade 1 vs 2	0.446 (0.295–0.675)	< 0.001	0.576 (0.371–0.894)	0.014		
FIB-4 < 3.25 vs ≥ 3.25	0.982 (0.664–1.452)	0.927				
BCLC stage B vs C	0.429 (0.208–0.885)	0.022	0.834 (0.362–1.922)	0.670	0.704 (0.307–1.617)	0.409
MVI (no vs yes)	0.849 (0.576–1.251)	0.408				
EHS (no vs yes)	0.709 (0.462–1.085)	0.113	1.048 (0.630–1.742)	0.858	1.163 (0.707–1.911)	0.552
AFP (ng/mL) < 20 vs ≥ 20 (ng/mL)	0.466 (0.308–0.705)	< 0.001	0.509 (0.325–0.795)	0.003		
ALBI grade 2 and AFP ≥ 20 ng/mL (yes vs no)	2.687 (1.826–3.954)	< 0.001			2.019 (1.338–3.047)	0.001
Albumin (g/dL)	0.468 (0.321–0.682)	< 0.001				
AST (U/L)	1.008 (1.004–1.011)	< 0.001				
ALT (U/L)	1.003 (1.000–1.006)	0.084				
Total bilirubin (mg/dL)	2.362 (1.518–3.676)	< 0.001				
PLT (10^9/L)	1.000 (0.996–1.004)	0.940				
INR	1.820 (1.061–3.121)	0.030	0.790 (0.383–1.626)	0.522	0.841 (0.427–1.659)	0.618
Sequential therapy (sorafenib and regorafenib vs sorafenib alone)	0.180 (0.109–0.298)	< 0.001	0.201 (0.117–0.345)	< 0.001	0.196 (0.116–0.332)	< 0.001
Locoregional therapy (yes vs no)	0.724 (0.495–1.060)	0.097	0.572 (0.376–0.871)	0.009	0.563 (0.376–0.842)	0.005

Table shading indicated that the variable has a confounding effect on other factors, and thus was not included in the multivariate analysis. Abbreviations: AFP—alpha-fetoprotein; ALBI—albumin–bilirubin; ALT—alanine aminotransferase; AST—aspartate aminotransferase; BCLC—Barcelona Clinic Liver Cancer staging; CI—confidence interval; EHS—extrahepatic spread; FIB-4—fibrosis index based on four factors; INR—international normalized ratio; MVI—macrovascular invasion; OS—overall survival; PLT—platelets.

Table S9. Results of univariate and multivariate Cox regression analyses of predictors of OS after failure of sorafenib therapy (subgroup without locoregional therapy, *n* = 78).

Patients without locoregional therapy after failure of sorafenib therapy (*n* = 78)						
			ALBI grade-based Model		Combined ALBI and AFP-based Model	
**Variables**	**Univariate Hazard Ratio (95% CI)**	***p* value**	**Multivariate Hazard Ratio (95% CI)**	***p* value**	**Multivariate Hazard Ratio (95% CI)**	***p* value**
Age (year)	0.985 (0.960–1.010)	0.243				
Male vs female	2.482 (0.979–6.295)	0.056	1.906 (0.660–5.502)	0.233	1.496 (0.570–3.923)	0.413
ALBI grade 1 vs 2	0.394 (0.195–0.795)	0.009	0.551 (0.262–1.161)	0.117		
FIB-4 < 3.25 vs ≥ 3.25	1.129 (0.628–2.028)	0.686				
BCLC stage B vs C	1.024 (0.317–3.309)	0.968				
MVI (no vs yes)	0.775 (0.431–1.392)	0.393				
EHS (no vs yes)	1.305 (0.685–2.488)	0.418				
AFP (ng/mL) < 20 vs ≥ 20 (ng/mL)	0.559 (0.313–0.998)	0.049	0.532 (0.286–0.990)	0.046		
ALBI grade 2 and AFP ≥ 20 ng/mL (yes vs no)	2.817 (1.589–4.994)	< 0.001			2.052 (1.102–3.822)	0.023
Albumin (g/dL)	0.411 (0.237–0.716)	0.002				
AST (U/L)	1.017 (1.010–1.025)	< 0.001				
ALT (U/L)	1.012 (1.004–1.020)	0.003				
Total bilirubin (mg/dL)	2.493 (1.320–4.708)	0.005				
PLT (10^9/L)	0.998 (0.985–1.010)	0.714				
INR	1.644 (0.940–2.875)	0.082	0.846 (0.411–1.741)	0.650	0.867 (0.433–1.735)	0.686
Sequential therapy (sorafenib and regorafenib vs sorafenib alone)	0.181 (0.091–0.358)	< 0.001	0.232 (0.113–0.479)	< 0.001	0.211 (0.104 –0.428)	< 0.001

Table shading indicated that the variable has a confounding effect on other factors, and thus was not included in the multivariate analysis. Abbreviations: AFP—alpha-fetoprotein; ALBI—albumin–bilirubin; ALT—alanine aminotransferase; AST—aspartate aminotransferase; BCLC—Barcelona Clinic Liver Cancer staging; CI—confidence interval; EHS—extrahepatic spread; FIB-4—fibrosis index based on four factors; INR—international normalized ratio; MVI—macrovascular invasion; OS—overall survival; PLT—platelets.

Table S10. Results of univariate and multivariate Cox regression analyses of predictors of OS after sorafenib therapy (all patients, *n* = 178).

**Patients with and without regorafenib therapy after failure of sorafenib therapy (*n* = 178)**						
			**ALBI grade-based Model**		**Combined ALBI and AFP-based Model**	
**Variables**	**Univariate Hazard Ratio (95% CI)**	***p* value**	**Multivariate Hazard Ratio (95% CI)**	***p* value**	**Multivariate Hazard Ratio (95% CI)**	***p* value**
Age (year)	0.986 (0.969–1.002)	0.092				
Male vs female	1.200 (0.731–1.970)	0.471				
ALBI grade 1 vs 2	0.499 (0.332–0.749)	0.001	0.740 (0.479–1.144)	0.176		
FIB-4 < 3.25 vs ≥ 3.25	1.118 (0.755–1.655)	0.577				
BCLC stage B vs C	0.292 (0.142–0.602)	0.001	0.532 (0.231–1.222)	0.137	0.498 (0.218–1.136)	0.098
MVI (no vs yes)	0.929 (0.634–1.362)	0.706				
EHS (no vs yes)	0.511 (0.332–0.786)	0.002	0.929 (0.565–1.529)	0.773	0.953 (0.582–1.561)	0.849
AFP (ng/mL) < 20 vs ≥ 20 (ng/mL)	0.466 (0.310–0.701)	< 0.001	0.644 (0.416–0.997)	0.049		
ALBI grade 2 and AFP ≥ 20 ng/mL (yes vs no)	2.339 (1.589–3.441)	< 0.001			1.504 (0.998–2.264)	0.051
Albumin (g/dL)	0.501 (0.343–0.732)	< 0.001				
AST (U/L)	1.010 (1.007–1.014)	< 0.001				
ALT (U/L)	1.006 (1.002–1.009)	0.002				
Total bilirubin (mg/dL)	2.576 (1.627–4.080)	< 0.001				
PLT (10^9/L)	1.001 (0.997–1.005)	0.719				
INR	2.151 (1.201–3.854)	0.010	1.025 (0.484–2.172)	0.949	1.087 (0.526–2.247)	0.821
Sequential therapy (sorafenib and regorafenib vs sorafenib alone)	0.153 (0.093–0.252)	< 0.001	0.201 (0.117–0.344)	< 0.001	0.190 (0.113–0.318)	< 0.001
Locoregional therapy (yes vs no)	0.740 (0.507–1.081)	0.119	0.738 (0.493–1.105)	0.140	0.739 (0.502–1.088)	0.125

Table shading indicated that the variable has a confounding effect on other factors, and thus was not included in the multivariate analysis. Abbreviations: AFP—alpha-fetoprotein; ALBI—albumin–bilirubin; ALT—alanine aminotransferase; AST—aspartate aminotransferase; BCLC—Barcelona Clinic Liver Cancer staging; CI—confidence interval; EHS—extrahepatic spread; FIB-4—fibrosis index based on four factors; INR—international normalized ratio; MVI—macrovascular invasion; OS—overall survival; PLT—platelets.

Table S11. Results of univariate and multivariate Cox regression analyses of predictors of OS after sorafenib therapy (subgroup without locoregional therapy, *n* = 78).

**Patients without locoregional therapy after failure of sorafenib therapy (*n* = 78)**						
			**ALBI grade-based Model**		**Combined ALBI and AFP-based Model**	
**Variables**	**Univariate Hazard Ratio (95% CI)**	***p* value**	**Multivariate Hazard Ratio (95% CI)**	***p* value**	**Multivariate Hazard Ratio (95% CI)**	***p* value**
Age (year)	0.985 (0.962–1.009)	0.232				
Male vs female	2.583 (1.018–6.552)	0.046	2.125 (0.700–6.448)	0.183	1.811 (0.681–4.815)	0.234
ALBI grade 1 vs 2	0.454 (0.225–0.914)	0.027	0.712 (0.329–1.543)	0.390		
FIB-4 < 3.25 vs ≥ 3.25	1.234 (0.689–2.211)	0.480				
BCLC stage B vs C	0.485 (0.149–1.577)	0.229	0.382 (0.113–1.292)	0.122	0.367 (0.110–1.221)	0.102
MVI (no vs yes)	1.180 (0.671–2.076)	0.566				
EHS (no vs yes)	0.768 (0.406–1.450)	0.415				
AFP (ng/mL) < 20 vs ≥ 20 (ng/mL)	0.601 (0.340–1.061)	0.079	0.604 (0.326–1.117)	0.108		
ALBI grade 2 and AFP ≥ 20 ng/mL (yes vs no)	2.186 (1.236–3.865)	0.007			1.610 (0.868–2.988)	0.131
Albumin (g/dL)	0.531 (0.309–0.912)	0.022				
AST (U/L)	1.016 (1.007–1.025)	< 0.001				
ALT (U/L)	1.011 (1.002–1.019)	0.012				
Total bilirubin (mg/dL)	2.664 (1.367–5.194)	0.004				
PLT (10^9/L)	1.001 (0.988–1.013)	0.902				
INR	2.002 (1.080–3.711)	0.027	1.199 (0.567–2.535)	0.635	1.253 (0.610–2.575)	0.539
Sequential therapy (sorafenib and regorafenib vs sorafenib alone)	0.215 (0.110–0.421)	< 0.001	0.258 (0.126–0.527)	< 0.001	0.245 (0.123–0.488)	< 0.001

Table shading indicated that the variable has a confounding effect on other factors, and thus was not included in the multivariate analysis. Abbreviations: AFP—alpha-fetoprotein; ALBI—albumin–bilirubin; ALT—alanine aminotransferase; AST—aspartate aminotransferase; BCLC—Barcelona Clinic Liver Cancer staging; CI—confidence interval; EHS—extrahepatic spread; FIB-4—fibrosis index based on four factors; INR—international normalized ratio; MVI—macrovascular invasion; OS—overall survival; PLT—platelets.
